# Successful Desensitization to the Radiocontrast Material Iohexol (Omnipaque™)

**DOI:** 10.7759/cureus.32356

**Published:** 2022-12-09

**Authors:** Abeer Saad, Asmaa S Mahdi, Iman Nasr

**Affiliations:** 1 Medicine, The Royal Hospital, Muscat, OMN

**Keywords:** oman, allergy, desensitization, iohexol (omnipaque), radiocontrast media

## Abstract

Adverse reactions to radiocontrast media (RCM) are rare and occur predominantly in association with intravenous administration but may also occur with intra-arterial and nonvascular injections (e.g., retrograde pyelography, intra-articular injections) of RCM. This article reports the case of a 52-year-old lady who was known to have amyloidosis secondary to rheumatoid arthritis and was on regular renal replacement therapy. She was under follow-up for regular angioplasties to manage the central vein stenosis that was affecting her right brachiocephalic arteriovenous fistula (AVF) and was referred to our Immunology service when she developed an allergic reaction after her AVF angioplasty (central venoplasty). Despite being dialysed immediately post-angioplasty, she complained of skin rash and itching with hoarseness of voice that developed almost six to eight hours post-angioplasty. We decided to arrange the iodinated non-ionic iso-osmolar contrast agent iodixanol (Visipaque™) for her instead, as it is known to be better tolerated in patients with reactions to Omnipaque™ due to its lower osmolarity as compared to Omnipaque™. However, since it was the first time to request this contrast in our hospital, it was not possible due to logistical reasons. It was necessary that our patient continued to undergo angioplasty every three months, however, she was developing more severe and earlier symptoms with each subsequent exposure to the radiocontrast medium. After her latest reaction of generalized itching and angioedema with shortness of breath during the procedure despite premedication, it was decided for her to undergo desensitization to Omnipaque™. In the absence of a published protocol for this, we used a protocol used for desensitization to Visipaque™. She showed an excellent response and completed her remaining angioplasties until Visipaque™ became available. Hence, desensitization to Omnipaque™ using the published protocol to Visipaque™ is likely to help patients allergic to Omnipaque™ or where Visipaque™ is not available or non-affordable in low/middle-income countries.

## Introduction

More than 70 million diagnostic radiographic examinations using radiocontrast media (RCM) are performed worldwide each year [1]. Adverse reactions to RCM are rare and occur predominantly in association with intravenous administration of RCM, but may also develop with intra-arterial and nonvascular injections of RCM [2]. Adverse reactions to RCM are divided into chemotoxic reactions and hypersensitivity reactions. Chemotoxic reactions e.g., vasovagal, arrhythmia, seizures, and renal toxicity are related to the chemical properties of RCM and are dependent upon dose and infusion rate [3]. Hypersensitivity reactions to RCM can occur in response to minute amounts of contrast agent. These reactions can be further subdivided into immediate (within one hour of administration) and delayed (from one hour to several days after administration) [3]. Herein, we report successful desensitization to iohexol (Omnipaque™), which has not been reported before for a haemodialysis patient requiring arteriovenous fistula (AVF) central venoplasty. 

## Case presentation

A 52-year-old lady, who was on follow-up for regular angioplasties to manage the central vein stenosis that was affecting her right brachiocephalic arteriovenous fistula (AVF), was referred to our immunology service when she developed an allergic reaction after her AVF angioplasty (central venoplasty). 

She was on thrice weekly regular haemodialysis since 2012 after being diagnosed with renal amyloidosis secondary to rheumatoid arthritis and initially underwent dialysis through central venous catheters (two tunneled internal jugular and subclavian vein catheters). In April 2013, she had a right brachiocephalic AVF created and a fistulogram in early 2014 showed central venous obstruction at the Superior Vena Cava junction and at the cephalic arch, thought to be a consequence of her history of multiple central venous catheters. Hence, she underwent AVF angioplasty for the first time in May 2014 using Omnipaque™. Subsequent follow-up angioplasties showed persistent stenosis, and she had a stent placed in September 2014. However, unfortunately, she then developed intra-stent stenosis. 

Since she previously had a left brachiocephalic AVF ligated and due to her rheumatoid fixed flexion hand and arm deformities, creating a new AVF was considered by her vascular surgeons to be technically challenging and owing to her persistent problem of central venous stenosis, any new AVF would be destined for the same fate as her current AVF, so it was decided between her vascular surgeon and interventional radiologist that she would need to be on a regular three monthly AVF angioplasty regimen to maintain the patency of her AVF. 

Unfortunately, in April 2015, despite being dialysed immediately post-angioplasty as was her routine each time post-procedure, she complained of skin rash and itching with hoarseness of voice that developed almost six to eight hours post-angioplasty. With each subsequent angioplasty, she continued to get worsening symptoms with generalized urticaria, angioedema, and chest tightness that occurred earlier than the previous times during the procedure requiring intraoperative hydrocortisone and chlorpheniramine maleate and therefore, in November 2015, she was referred to the immunologist for assessment. The radiocontrast material used was Omnipaque™ which was the only radiocontrast material in our radiology department.

As there was no alternative to Omnipaque™ at that time, it was decided in the management strategy to administer her a standard pre-medication protocol consisting of cetirizine 10 mg and ranitidine 300 mg at seven hours and then one hour pre-procedure along with intramuscular hydrocortisone 100 mg, at 13, seven and one hour pre-procedure along with intravenous methylprednisolone 50 mg one hour prior to the procedure in daycare. Despite this, she continued to have allergic symptoms that started with facial flushing, itching, and erythematous urticarial rash that spread to involve her whole body with severe itching persisting for a couple of days post-procedure.

Consequently, we decided to arrange for her the iodinated non-ionic iso-osmolar contrast agent iodixanol (Visipaque™) instead, as it is known to be better tolerated in patients with reactions to Omnipaque™ due to its lower osmolarity as compared to Omnipaque™​​​​​​​ but unfortunately it was not available at our hospital and required many months before it would become available. Since it was necessary for her to undergo angioplasty every three months, desensitization to Omnipaque™ was considered. On searching the literature, we could not find any desensitization protocol for Omnipaque™ but found a case report of successful desensitization to the lower osmolar RCM, Visipaque™. Due to the urgency of the angioplasty, we devised a rapid intravenous desensitization protocol for Omnipaque™ RCM using the protocol used in Visipaque™ desensitization.

**Figure 1:**
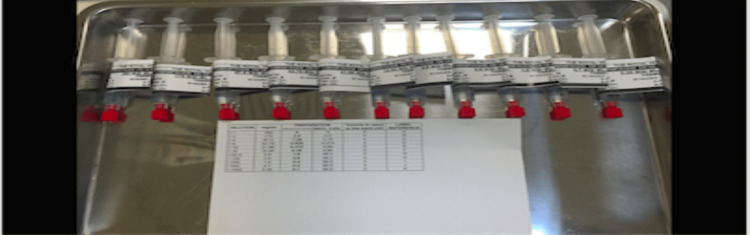
Omnipaque™ contrast desensitization protocol's preparation

The procedure was performed in our daycare unit under close monitoring with vitals frequently checked throughout the desensitization. The pretreatment regimen was augmented, which included the following: hydrocortisone 100 mg intramuscularly (seven hours and one hour before the procedure), chlorpheniramine maleate 4 mg, ranitidine 300 mg orally, and methylprednisolone 100 mg intravenously (all one hour before the initiation of intravenous desensitization). The desensitization protocol was initiated two hours before angiography. We gradually administered graded solutions of Omnipaque™ starting from a dilution of 1:1000 till finally reaching 1:1. This last dose was given by the interventional radiologist during the procedure (our protocol is detailed in Table 1 and Figure 1). 

**Table 1:** Omnipaque™ contrast desensitization protocol (adopted from the desensitization protocol to Visipaque™) * AVF angioplasty proceeded after dose 10, with a total of 20 mL of Omnipaque™.

Dose no.	Dilution	Concentration (mg/ml)	Volume to administer (mL)	Time of administration (min)
1	1:1000	0.35	5	0
2	1:500	0.7	5	10
3	1:250	1.4	5	20
4	1:125	2.8	5	30
5	1:62.5	5.6	5	40
6	1:32	10.94	5	50
7	1:16	21.88	5	60
8	1:8	43.75	5	70
9	1:4	87.5	5	90
10*	1:2	175	5	120
11	1:1	350	-	-

The patient reported mild perioral flushing and itching with mild lower lip swelling at step 8 (strength 1:8) that resolved immediately with 100 mg of intravenous hydrocortisone and 10 mg of intravenous chlorpheniramine maleate. The desensitization protocol was continued with no further symptoms. The AVF central venoplasty was completed using 20 mL of Omnipaque™ in total, with complete hemodynamic stability and no untoward symptoms throughout the procedure. After the procedure, our patient underwent haemodialysis and was stable. She was discharged in a good condition with no reported delayed reactions. The desensitization was successfully repeated again at the next appointment. Subsequently, we were able to procure Visipaque™ for her and she had been undergoing her angioplasties using this with premedication, not developing any allergic symptoms and thus not requiring desensitization.

## Discussion

Symptoms of RCM reaction include cutaneous eruptions, urticaria, angioedema, and several uncommon reactions, such as erythema multiforme, drug eruption, and Stevens-Johnson syndrome [3]. Immediate hypersensitivity reactions (IHRs) to RCM can be clinically identical to severe immunoglobulin E (IgE)-mediated anaphylaxis [4]. The pathophysiology of IHRs is believed to be non-IgE-mediated in most cases, although in a small percentage, it may involve IgE [5]. Several mechanisms have been proposed. Enzyme induction leads to the release of vasoactive substances, including serotonin and histamine, the activation of a physiological cascade and the complement system [5]. The ability of a 'psuedoallergen' like RCM to activate the complement system results in excessive anaphylatoxin generation in blood. These reactions have been called "Complement activation-related pseudoallergy" (CARPA) [6].

RCM are categorized by osmolality: firstly, high-osmolal contrast material (HOCM) agents (osmolalities ≥ 1400 mosm/kg); secondly, low-osmolal contrast material (LOCM) agents (osmolalities between 500 to 900 mosm/kg such as iohexol Omnipaque™); thirdly, iso-osmolal agents, which are isotonic relative to serum (approximately 290 mosmol/kg, e.g., iodixanol Visipaque™). Iso-osmolal agents have a lower osmolality than "low-osmolal" agents [7]. The use of nonionic LOCM agents for all intravascular procedures has become a widespread practice in most centres, especially with the low difference in cost between LOCM and HOCM [7]. The rate of IHRs is significantly lower with the use of the iso-osmolal RCM such as iodixanol (Visipaque™) as compared to the nonionic LOCM agents [7,8]. 

In the absence of a history of a reaction to RCM, skin testing and intravenous test dosing of RCM is not helpful in the prediction or prevention of IHRs and are therefore not recommended as fatal reactions to usual doses of RCM have occurred in patients who were tested negative on skin testing and tolerated test doses of that agent [9,10]. 

The widespread use of LOCM for all intravascular procedures has largely obviated the need for premedication. Patients with a history of IHR to RCM are at an increased risk for recurrent IHR if re-exposed to that RCM. In such cases, a different contrast agent with lower osmolal content along with premedication is recommended. An allergy evaluation may be warranted if the past reaction was severe. Premedication has been used to help in reducing recurrent IHRs. Although the optimal approach has not been determined and though they may reduce IHRs, they cannot fully prevent severe reactions and may, sometimes, result in breakthrough reactions [10,11]. The use of RCM in diagnostic and therapeutic angiography and vascular interventions is limited by nephrotoxicity and allergic reactions. Non-iodine-based RCM is used in cases of IHRs to the iodinated RCM such as carbon monoxide and gadolinium but each has its own limitations and complications and may not be ideal. In addition, some centers may only have LOCM with no access to an iso-osmolal one. Hence, there is a pressing need to have an alternative management strategy whereby the iodinated RCM can be administered safely despite known hypersensitivity to them. This is where rapid desensitization therapy becomes important [11,12].

Rapid desensitization is a gradual reintroduction of the first-line medications that the patient is allergic to by providing a temporary tolerance to the drug. It's used only where no similarly effective alternative is available [12,13]. This procedure has been proven effective for IgE-mediated reactions where in vitro studies were done to prevent mast cell activation [12]. It was noted that repetitive administration of increasing allergen doses over the course of a few hours during the build-up phase led to the exhaustion of stored mediators due to repetitive stimulation and release [13].

Few successful cases of desensitization to RCM have been reported, mostly using iodixanol (Visipaque™) in patients who developed IHRs despite premedication [4,14,15]. One in an 81-year-old female with anaphylaxis to iodixanol despite augmented pre-treatment and an 82-year-old male who underwent a successful graded dose challenge to iodixanol after having delayed anaphylaxis to iohexol. In our case, the desensitization was successful, and the patient underwent angioplasty uneventfully. The desensitization was successfully repeated again on the next appointment. Subsequently, we were able to procure Visipaque™ for her and she has been undergoing her angioplasties using it as the radiocontrast medium with premedication with no symptoms and without the need for desensitization.

## Conclusions

This novel approach to RCM hypersensitivity management provides hope that desensitization may play a greater role in the management of patients allergic to HOCM, where Visipaque™ is not available and/or not affordable in low/middle-income countries. A rapid desensitization protocol just before angiography was helpful and allowed us to intervene successfully for a patient with multiple severe non-IgE-mediated anaphylactic reactions to the current contrast medium despite premedication.
